# Systemic cardiovascular and carotid baroreflex support of arterial pressure during recovery from passive heat stress in young and older adults

**DOI:** 10.14814/phy2.70554

**Published:** 2025-09-10

**Authors:** Emily A. Larson, Brendan W. Kaiser, Emma L. Reed, Brandon M. Gibson, Kieran S. S. Abbotts, W. Larry Kenney, Christopher T. Minson, John R. Halliwill

**Affiliations:** ^1^ Department of Human Physiology University of Oregon Eugene Oregon USA; ^2^ Department of Kinesiology The Pennsylvania State University University Park Pennsylvania USA

**Keywords:** blood pressure, body temperature regulation, hemodynamics, hyperthermia, thermoregulation

## Abstract

We evaluated the systemic cardiovascular and carotid baroreflex support of arterial pressure during recovery from whole‐body, passive heating in young and older adults. Supine mean arterial pressure (MAP), cardiac output (Q; acetylene washin), systemic vascular conductance (SVC), heart rate (HR), and stroke volume (SV) were evaluated in 16 young (8F, 18–29 years) and nine older (6F, 61–73 years) adults at normothermic baseline and for 60‐min passive heating and 120‐min normothermic recovery. Externally applied neck pressure was used to evaluate HR, brachial vascular conductance, and MAP responses to carotid baroreceptor unloading. MAP was reduced with heating in young adults (*p* < 0.001) and similar to baseline within 30 min recovery in both age groups. Heating‐induced Q, SVC, HR, and SV responses returned to baseline within 60 min recovery in both age groups. Heating attenuated HR and brachial vascular responses to neck pressure in young adults (both *p* < 0.01), but these responses did not differ from baseline throughout recovery in either age cohort. MAP responses to neck pressure were not impacted by heating in either age group but increased after 90 min recovery in young adults (*p* < 0.05). The systemic cardiovascular and carotid baroreflex responses accompanying passive heating do not persist beyond 30 min of post‐heating recovery in young or older adults.

## 1 INTRODUCTION

Since Fitzgerald's early observation that daily jogging improved his labile hypertension (Fitzgerald, 1981), decades of research have established the post‐exercise recovery period as a discrete physiological phenomenon distinct from the stressed and rested state that promotes clinically relevant reductions in blood pressure (Carpio‐Rivera et al., 2016) and alterations in central and peripheral cardiovascular control (Halliwill, 2001; Halliwill et al., 1996, 2013; McCord & Halliwill, 2006). Much like exercise, acute whole‐body, passive heat stress elicits widespread thermoregulatory, cardiovascular, and autonomic adjustments and may promote a distinct post‐stress recovery period. However, in stark contrast to the post‐exercise recovery period, few studies have examined the physiology of post‐heating recovery.

Advancing age alters the thermoregulatory and cardiovascular responses to acute heat stress (Minson et al., 1998, 1999) and these alterations may potentiate age‐based differences in the systemic cardiovascular regulation of arterial pressure in the post‐heating recovery period. Studies examining arterial pressure and systemic cardiovascular responses following acute heat stress among healthy, young adults have yielded conflicting findings. Francisco et al. (2021) demonstrated sustained hypotension for 1 h following seated chest‐level hot water immersion in healthy, young adults, which was comparable to the reduction in blood pressure that followed a single session of moderate intensity aerobic exercise (post‐exercise hypotension). In contrast, studies utilizing seated lower limb hot water immersion (Romero et al., 2017) and supine leg heating via water‐perfused suit (Engelland et al., 2020b) have noted that mean arterial pressure returns to baseline values within 30 min of supine post‐heating recovery in young adults. Sustained hypotension following passive heating may be more consistent among healthy, older adults and has been demonstrated 30 min into supine recovery following various heating modalities, including lower limb hot water immersion (Romero et al., 2017), leg heating via water‐perfused suit (Engelland et al., 2020b), and waist‐level hot water immersion (Thomas et al., 2017).

While this work provides valuable insight into the post‐heating recovery period, several gaps in knowledge remain. First, previous studies examining post‐heating recovery in both young and older adults (Engelland et al., 2020b; Romero et al., 2017) utilized a mild, lower body heating modality (peak Δ intestinal temperature + 0.4–0.8°C) and may not reflect recovery responses following more intensive, whole‐body heating regimens. Additionally, previous studies completed in both young and older adults conducted assessments at a single time point, 30 min into post‐heating recovery amidst elevations in core and skin temperature. As a result, the dynamic changes that may occur throughout the post‐heating recovery period as core and/or skin temperature recover and the total duration of these responses are unknown.

Moreover, while considerable investigation has focused on evaluating baroreflex function during passive heating (Crandall, 2000; Cui et al., 2002a, 2002b; Krnjajic et al., 2016; Yamazaki et al., 1997), no studies to our knowledge have interrogated baroreflex function during post‐heating recovery. Engelland et al. (2020b) demonstrated that muscle sympathetic nerve activity did not differ from baseline values following heating in older adults despite an ~7 mmHg reduction in arterial pressure, suggesting that post‐heating recovery alters baroreflex control of the heart and/or vasculature. Importantly, this hypotension and relative sympathoinhibition was not demonstrated among young adults, suggesting that advancing age may modify baroreflex control in the post‐heating recovery period.

This study aimed to address these gaps in knowledge and compare the systemic cardiovascular and carotid baroreflex support of arterial pressure during 2 hours of normothermic recovery from whole‐body, passive heat stress in healthy, young and older adults. We predicted that post‐heating hypotension would occur in both age groups and be accompanied by sustained vasodilation and attenuated carotid baroreflex control of the peripheral vasculature.

## 2 METHODS

### 2.1 Ethical approval

This study was approved by the Institutional Review Board at the University of Oregon. Prior to participation, all participants provided oral and written informed consent as set forth by the Declaration of Helsinki.

### 2.2 Participants

Sixteen young (8 self‐reported women, 8 self‐reported men) and nine older (6 self‐reported women, 3 self‐reported men) volunteers participated in this study. All individuals were healthy with no history of cardiovascular disease and not taking prescription medications other than hormonal contraceptives. Individuals were excluded from participation in the study if they had Stage 2 hypertension (≥140 mmHg systolic blood pressure and/or ≥90 mmHg diastolic blood pressure), were underweight or obese (body mass index ≤18.5 or ≥35 kg·m^−2^), were endurance training (moderate aerobic exercise ≥60 min/session on ≥5 days per week), or participating in a regular heat therapy regimen (whole‐body heating ≥30 min/session on ≥3 days per week).

Females were studied under low hormone conditions when possible: during the early follicular phase of the menstrual cycle, placebo phase of oral contraceptive use, and/or in the absence of hormone replacement therapy. Before screening and experimental study days, participants abstained from over‐the‐counter medications for >24 h, heavy exercise and heat stress for >24 h, alcohol and caffeine for >12 h, and food for 4 h (screening visit) or 2 h (study visit). Participants on a daily aspirin regimen refrained from aspirin for 1 week before participating in the experimental session. Adequate hydration was evaluated in all participants prior to each experimental session by a first morning urine‐specific gravity of less than 1.024. If urine‐specific gravity was greater than 1.024, participants drank 3 mL water/kg of body weight before beginning the protocol. This urine sample was used to conduct a pregnancy test on participants of child‐bearing potential.

### 2.3 Experimental protocol

The screening/familiarization visit consisted of health history and physical activity (IPAQ‐SF) questionnaires, height and weight measurement, body composition estimation based on skinfold thickness measured at three standard locations (men: chest, abdomen, thigh; women: triceps, suprailiac, thigh) (Jackson et al., 1980; Jackson & Pollock, 1978), and seated resting blood pressure assessment (Whelton et al., 2018). Auscultation of the carotid arteries was performed, and participants were excluded from further participation in the study if vascular sounds suggestive of reduced or turbulent carotid arterial blood flow were detected. Vascular ultrasound was used to determine the location of the carotid bifurcation, and the distance from the carotid bifurcation to the angle of the mandible was measured to ensure the neck collar would encompass the bifurcation. Participants completed several rounds of familiarization with the neck pressure technique and were excluded from the study if an adequate fit of the neck collar could not be obtained.

The experimental study visit began between 0700 and 1000 and consisted of a normothermic baseline period, 60 min whole‐body, passive heating via a water perfused suit, and 2 h normothermic recovery. Upon arrival to the laboratory and initial instrumentation, participants rested in the supine position and donned a two‐piece water perfused suit (Med‐Eng Systems, Ottawa, Canada) that covered the entire body except for the head, hands, feet, and calves. A water circulating bath (HT CIRC 7 L AD, Polyscience, Niles, Illinois) was used to perfuse 34°C water through the suit at baseline. Following at least 20 min of supine rest, baseline measures were completed over the course of ~1 h. Participants were then wrapped in mylar thermal and cotton blankets and wore a winter cap while 50°C water was perfused through the suit for 60 min. If rectal temperature increased by 1°C, the temperature of the water was decreased to 45°C. In all participants, the blankets and hat were removed prior to the neck pressure measurements during the passive heating intervention. After all passive heating measurements were completed, 34°C was circulated through the suit for 2 h of normothermic recovery. Fluid consumption (3 mL water/kg of body weight) was restricted to the heating portion of the experimental visit. Total sweat loss was estimated by measuring fluid consumption, urine losses, and dry nude body weight at the beginning and end of the experimental session.

### 2.4 Measurements

#### 2.4.1 Thermal

Rectal and skin temperature were determined at normothermic baseline, every 10 min during passive heating, and every 30 min during the 2 h normothermic recovery period. A rectal thermistor probe was inserted ~10 cm past the anal sphincter (YSI series 400: Yellow Spring Instruments, Yellow Springs, OH). Six thermochron temperature loggers (DS1922L, 11‐bit resolution, OnSolution Pty Ltd., NSW, AU) were placed at standard locations, and mean skin temperature was calculated as the weighted average of these six assessment sites (chest 22%, upper back 19%, lower back 19%, abdomen 14%, thigh 14%, calf 11%) (Gagnon et al., 2015). Mean body temperature was estimated as the weighted average of rectal (90%) and mean skin temperature (10%).

#### 2.4.2 Central hemodynamic

Arterial pressure (automated auscultatory via Tango+, SunTech Medical, Raleigh, NC), heart rate (Lead II; Cardiocap 5, Datex‐Ohmeda, St. Louis, MO), and cardiac output were measured at baseline and every 30 min during passive heating and normothermic recovery. Cardiac output was measured non‐invasively using the open circuit acetylene washin method (Johnson et al., 2000). Participants breathed a gas mixture containing 0.6% acetylene, 9.0% helium, 20.9% oxygen, and balance nitrogen for 8–10 breaths via a two‐way non‐rebreathing valve attached to a pneumatic sliding valve. During the washin phase, breath‐by‐breath acetylene and helium uptake were measured by a respiratory mass spectrometer (MGA 1100; MA Tech Services, Inc.; Saint Louis, MO, USA), and total volume was measured via a pneumotach (Series 1110, Hans Rodolph, Kansas City, MO, USA) linearized and calibrated using test gases before each testing day. Stroke volume (mL) was calculated as cardiac output divided by heart rate, and systemic vascular conductance (mL/min/mmHg) was calculated as cardiac output divided by mean arterial pressure.

#### 2.4.3 Calf and skin blood flow

Calf blood flow (mL/min/dL tissue) was measured at baseline and every 30 min during passive heating and normothermic recovery using venous occlusion plethysmography on the right calf. Instrumentation included a venous occlusion cuff placed just above the knee, an arterial occlusion cuff placed around the ankle (A.T.S. Disposable Cuffs, Zimmer Biomet, Warsaw, IN), and a mercury‐in‐Silastic strain gauge (Hokanson Inc., Bellevue, WA) placed around the widest girth of the calf and connected to a plethysmograph (EC6 Plethysmograph, Hokanson Inc., Bellevue, WA). Changes in calf volume during transient inflation (60 mmHg for 7.5 s out of every 15 s) of the venous occlusion cuff provided one measurement of calf blood flow every 15 s. The arterial occlusion cuff around the ankle was inflated to 250 mmHg 1 min prior to calf blood flow measures. Calf vascular conductance (mL/min/dL tissue/mmHg) was calculated as calf blood flow divided by mean arterial pressure. Skin blood flow was measured using laser Doppler flowmetry at baseline, every 10 min during passive heating, and every 30 min during normothermic recovery on the left calf. Two single‐point (MP12; Moor Instruments, Axminster, UK) and two integrated laser Doppler probes (MP1/7‐V2; Moor Instruments, Axminster, UK) were placed flush against and perpendicular to the surface of the skin of the calf. The single point probes were seated within local heating units (SH02 Skin Heater/Temperature Monitor; Moor Instruments, Axminster, UK) that were heated to 43.5°C at a rate of 0.1°C/s after 90 min of normothermic recovery to obtain maximal flux (Minson, 2010). Cutaneous vascular conductance was calculated as skin red blood cell flux divided by mean arterial pressure. For the two single point probe sites where local heating was completed, cutaneous vascular conductance values are presented as a percentage of maximum cutaneous vascular conductance.

#### 2.4.4 Carotid baroreflex assessments

Neck pressure was applied to assess carotid baroreflex function at normothermic baseline, at the end of passive heating, and every 30 minutes during normothermic recovery. During these time points, participants were instrumented with an external neck collar that enclosed the anterior two‐thirds of the neck and was sealed against the mandible, clavicles, and sternum. The neck collar was connected to a bellows pressure controller (PPC‐1000, Engineering Development Laboratory, Inc., Newport News, VA) and interfaced with custom software to deliver R‐wave activated 5 s bouts of 50 mmHg neck pressure. Each neck pressure stimulus was administered during a voluntary end expiration apnea (Eckberg et al., 1980) which was initiated 3 s prior to neck pressure application and maintained for 7 s following the neck pressure application. This process was repeated four to six times, with each neck pressure application preceded by at least 1 min of paced breathing at 0.25 Hz (Buck et al., 2015; Pellinger & Halliwill, 2007).

During each neck pressure application, heart rate and beat‐by‐beat arterial pressure (Nexfin, BMEYE, Amsterdam, Netherlands) were monitored at 250 Hz (Windaq, Dataq Instruments, Akron, OH). Brachial artery blood flow was measured via duplex ultrasonography (Phillips iE33 ultrasound with 9 MHz linear‐array vascular probe, Andover, MA, USA) with an insonation angle of 60°. A pressure cuff was placed around the wrist and inflated to 250 mmHg 1 min prior to ultrasound measurements. Ultrasound measurements were made in the distal third of the upper arm, and probe placement location was marked to ensure the same area was imaged for repeated measurements. A 5‐s B‐mode recording was completed before each set of neck pressure trials, and automated wall tracking software (Vascular Research Tools 5; Medical Imaging Applications LLC, Coralville, IA) was used to determine brachial artery diameter (cm). The retrograde and anterograde audio signal output from the Doppler ultrasound was Fourier transformed to generate a continuous analog signal of mean blood velocity (cm/s) and recorded at 250 Hz. Blood flow velocity was multiplied by artery cross‐sectional area to determine brachial blood flow (mL/min) and brachial vascular conductance (mL/min/mmHg) was calculated as blood flow divided by mean arterial pressure.

For each neck pressure trial the average heart rate, mean arterial pressure, and brachial vascular conductance determined over the 3 s period of end‐expiration apnea prior to neck pressure application was used to calculate neck pressure‐induced changes in these variables. Cardiac baroreflex responsiveness was the peak heart rate response 1–7 s after the onset of neck pressure, integrated carotid baroreflex responsiveness was the peak mean arterial pressure response 2–9 s after the onset of neck pressure, and peripheral vascular carotid baroreflex responsiveness was the nadir of the brachial vascular conductance response 3–12 s after the onset of neck pressure. These time windows encapsulated the peak carotid baroreflex‐mediated responses to neck pressure application in both age groups (Fisher et al., 2009; Pellinger & Halliwill, 2007). Changes in brachial vascular conductance with applied neck pressure were used as an index of peripheral vascular responsiveness and are presented as a percentage change from the prevailing brachial vascular conductance prior to neck pressure application at each time point (Buckwalter & Clifford, 2001; Keller et al., 2003, 2004).

### 2.5 Data and statistical analysis

Rectal temperature, heart rate, calf blood flow, and skin blood flow data were recorded at 250 Hz (Windaq, *Dataq* Instruments, Akron, OH) and skin temperature was recorded at 0.02 Hz (eTemperature, OnSolution Pty Ltd., New South Wales, AU). Average values for these variables were determined across two 5 min bins at normothermic baseline and across a single 2 min bin during passive heating or normothermic recovery time points. Statistical analyses were conducted using GraphPad Prism 10.4.1. Participant characteristics data are presented as means and standard deviations, and all other data are presented as means and 95% confidence intervals. Paricipant characteristics and total sweat loss data were analyzed using unpaired, two‐tailed *t*‐tests. Inferences were drawn from two‐way mixed‐effects models (for measurements across time and between age groups) using Dunnett's multiple comparisons test for changes over time versus baseline within each age group and Šidák's multiple comparisons test to compare between groups at the same time point, with each corrected for multiplicity. Alpha was set at 0.05 for all statistical inferences including familywise error rates.

## 3 RESULTS

### 3.1 Participant characteristics

Participant characteristics for the 16 young (8 self‐reported men, 8 self‐reported women) and nine older adults (3 self‐reported men, 6 self‐reported women) who participated in the study are presented in Table 1. Young and older adults had similar estimated sweat losses during the experimental session [Young: −0.7 (−0.5, −0.9) kg, Older: −0.6 (−0.3, −0.9) kg, *p* = 0.46], which were equivalent to a sweat loss of −1.0 (−0.8, −1.3)% and −0.8 (−0.4, −1.2)% of initial body weight in young and older adults, respectively (*p* = 0.33).

**TABLE 1:** Participant characteristics.

	Young	Older
*n*	16 (8M, 8F)	9 (3M, 6F)
Age (year)	22 ± 4	65 ± 4*
Height (cm)	169 ± 13	171 ± 9
Weight (kg)	69 ± 15	72 ± 20
Body mass index (kg/m^2^)	24 ± 4	24 ± 5
Body fat percentage (%)	16 ± 10	23 ± 6
IPAQ‐SF MET‐min per week	2072 ± 952	3130 ± 2680
Systolic blood pressure (mmHg)	116 ± 7	114 ± 9
Diastolic blood pressure (mmHg)	71 ± 5	68 ± 6
Heart rate (bpm)	71 ± 10	60 ± 9
Left carotid bifurcation distance (cm)	3.1 ± 0.9	3.5 ± 0.8
Right carotid bifurcation distance (cm)	3.1 ± 0.7	3.7 ± 0.6*

*Note*: Values are means ± standard deviation. Data were compared between groups using an unpaired *t*‐test.

* *p* < 0.05 young versus older.

### 3.2 Temperature

**FIGURE 1:**
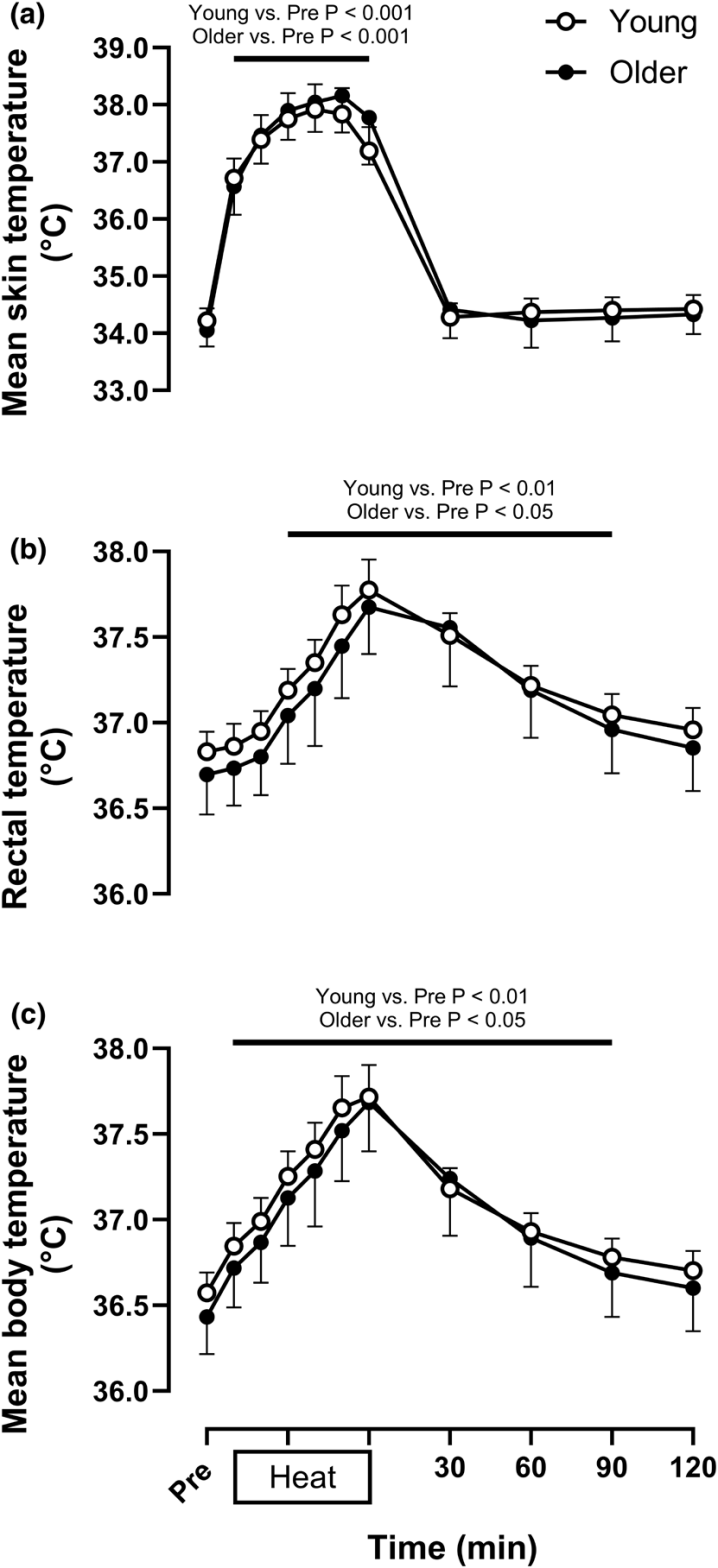
Mean skin, rectal, and body temperature at normothermic baseline (Pre), every 10 min during 60 min of passive heating, and every 30 min during 2 h of normothermic post‐heating recovery in young (open circles, *n* = 16) and older individuals (filled circles, *n* = 9). Values are means ±95% confidence intervals. Inferences were drawn from two‐way mixed‐effects models (age X time) using Dunnett's multiple comparisons test for changes over time versus baseline within each age group and Šidák's multiple comparisons test to compare between groups at the same time point.

As displayed in Figure 1, there were no differences in skin temperature [Young: 34.2 (34.0, 34.4)°C, Older: 34.0 (33.8, 34.3)°C, *p* > 0.99], core temperature [Young: 36.8 (36.7, 36.9)°C, Older: 36.7 (36.5, 37.0)°C, *p* > 0.99], or mean body temperature [Young: 36.6 (36.5, 36.7)°C, Older: 36.5 (36.3, 36.7)°C, *p* > 0.99] between age groups at baseline. By the final measurements during passive heating (50 min), skin [Young: +3.6 (+3.1, +4.1)°C, Older: +4.3 (+3.6, +5.0)°C, *p* = 0.15], core [Young: +0.8 (+0.7, +0.9)°C, Older: +0.8 (+0.6, +0.9)°C, *p* > 0.99], and mean body temperature [Young: +1.1 (+0.9, +1.2)°C, Older: +1.1 (+1.0, +1.3)°C, *p* > 0.99] were similarly elevated in young and older adults. While skin temperature did not differ from baseline values throughout the recovery period while 34°C water was circulated through the suit, core and mean body temperature remained elevated through 90 min of post‐heating recovery in both age groups.

### 3.3 Central hemodynamics

**FIGURE 2:**
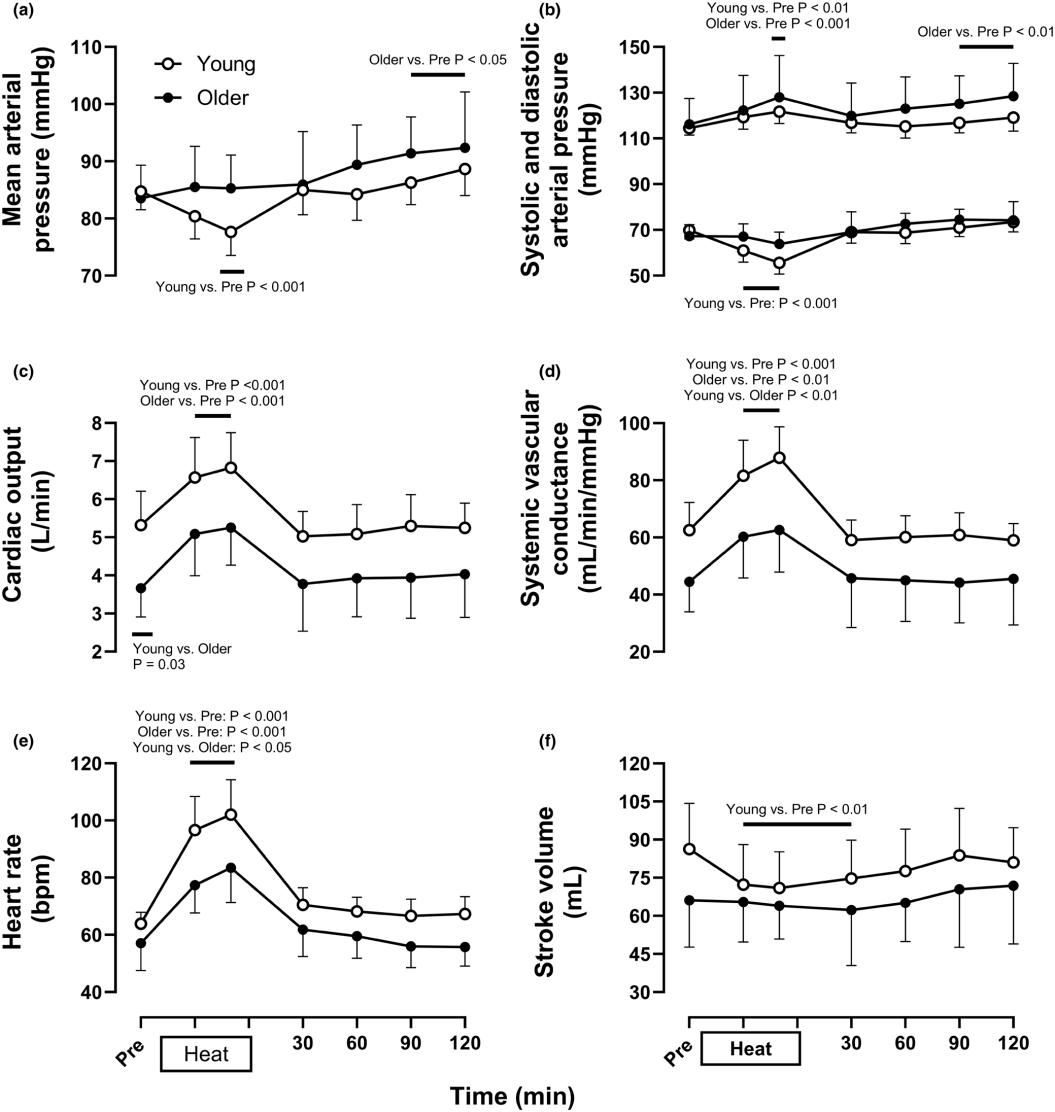
Mean (a), systolic and diastolic (b) arterial pressure, cardiac output (c), systemic vascular conductance (d), heart rate (e), and stroke volume (f) at normothermic baseline (Pre) and every 30 min throughout 60 min of passive heating and 2 h of normothermic post‐heating recovery in young (open circles, *n* = 16) and older individuals (filled circles, *n* = 9). Values are means ±95% confidence intervals. Inferences were drawn from two‐way mixed‐effects models (age X time) using Dunnett's multiple comparisons test for changes over time versus baseline within each age group and Šidák's multiple comparisons test to compare between groups at the same time point.

Central hemodynamic measures during whole‐body, passive heating and normothermic recovery are presented in Figure 2. There were no differences in mean [Young: 85 (82, 88) mmHg, Older: 83 (78, 88) mmHg, *p* > 0.99], systolic [Young: 115 (111, 118) mmHg, Older: 115 (105, 125) mmHg, *p* > 0.99], or diastolic blood pressure [Young: 70 (66, 74) mmHg, Older: 68 (63, 72) mmHg, *p* > 0.99] between age groups at baseline. Systolic blood pressure increased similarly during passive heating in both age groups [Young: +7 (+3, +11) mmHg, Older: +12 (+5, +20) mmHg, *p* = 0.47]. Diastolic blood pressure was reduced to a greater extent in young compared to older adults [Young: −14 (−19, −10) mmHg, Older: −3 (−6, −1) mmHg, *p* < 0.01]. As a result, mean arterial pressure was reduced with passive heating in young adults, but maintained near baseline values in older adults [Young: −7 (−11, −3) mmHg, Older: +2 (0, +4) mmHg, *p* < 0.01]. Mean arterial pressure was similar to baseline through 120 min of post heating recovery in young adults and 60 min of post‐heating recovery in older adults and increased relative to baseline levels in older adults thereafter. This was due to increases in systolic blood pressure from 90 min of recovery onward in older adults.

Cardiac output [Young: 5.3 (4.4, 6.2) L/min, Older: 3.6 (2.9, 4.3) L/min, *p* = 0.03] was greater in young versus older adults at baseline, while systemic vascular conductance [Young: 63 (53, 72) mL/min/mmHg, Older: 44 (34, 53) mL/min/mmHg, *p* = 0.05], heart rate [Young: 64 (60, 68) bpm, Older: 57 (49, 65) bpm, *p* = 0.82] and stroke volume [Young: 86 (68, 104) mL, Older: 66 (48, 84) mL, *p* = 0.48] did not differ between age groups. Cardiac output [Young: +1.5 (+1.0, +2.0) L/min, Older: +1.6 (+1.1, +2.2) L/min, *p* > 0.99] and systemic vascular conductance [Young: +25 (+19, +32) mL/min/mmHg, Older: +18 (+11, +26) mL/min/mmHg, *p* = 0.63] increased similarly during passive heating between age groups. Cardiac output and systemic vascular conductance did not differ from baseline values throughout post‐heating recovery in both age groups. Elevations in cardiac output with heating were supported by increases in heart rate in both young and older adults [Young: +38 (+28, +49) bpm, Older: +27 (+19, +35) bpm, *p* = 0.08]. Heart rate did not differ from baseline values throughout post‐heating recovery in either age group. Stroke volume was reduced below baseline values in young adults with heating [Young: −15 (−23, −8) mL], but was near baseline values in older adults [Older: −2 (−15, +11) mL, *p* = 0.15]. Stroke volume remained below baseline values for 30 min of post‐heating recovery in young adults but was similar to baseline values throughout post‐heating recovery in older adults.

### 3.4 Calf and skin blood flow

**FIGURE 3:**
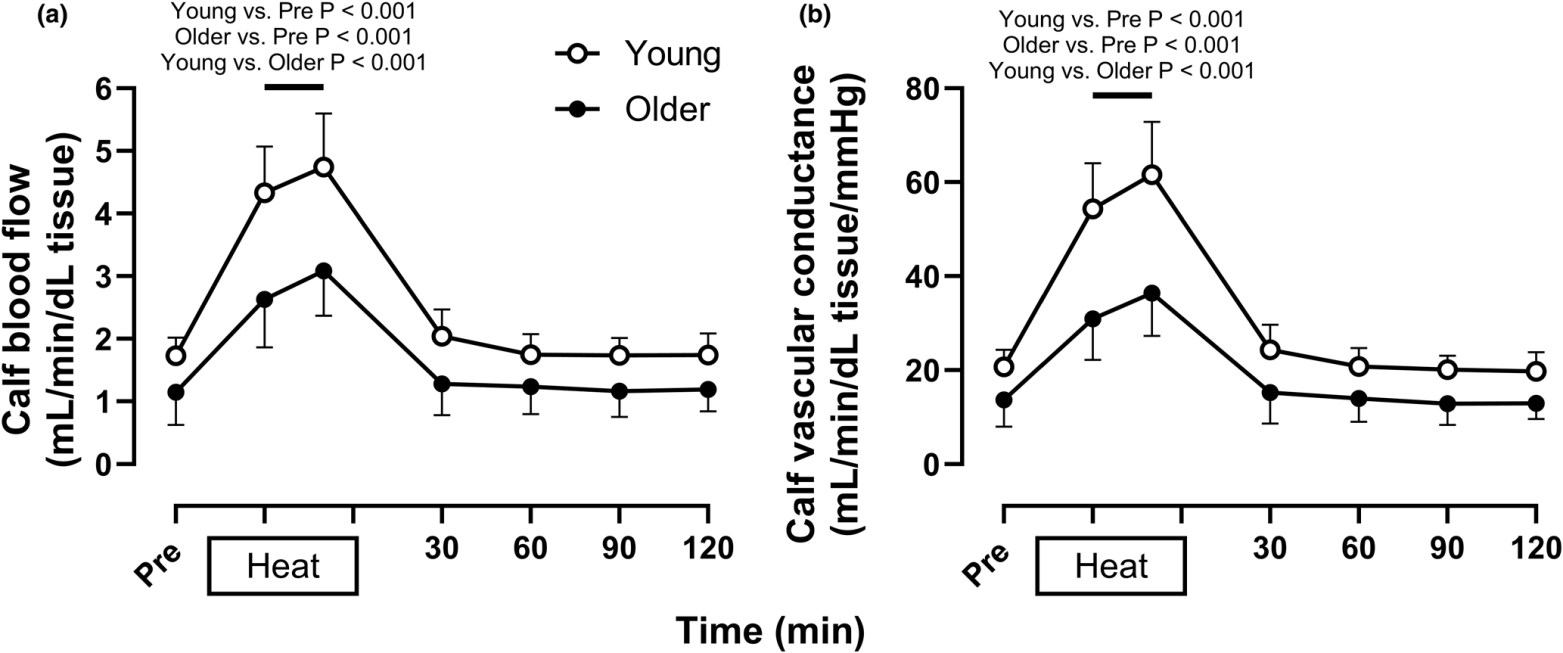
Calf blood flow (a) and vascular conductance (b) at normothermic baseline (Pre) and every 30 min throughout 60 min of passive heating and 2 h of normothermic post‐heating recovery in young (open circles, *n* = 16) and older individuals (filled circles, *n* = 9). Values are means ±95% confidence intervals. Inferences were drawn from two‐way mixed‐effects models (age X time) using Dunnett's multiple comparisons test for changes over time versus baseline within each age group and Šidák's multiple comparisons test to compare between groups at the same time point.

**FIGURE 4:**
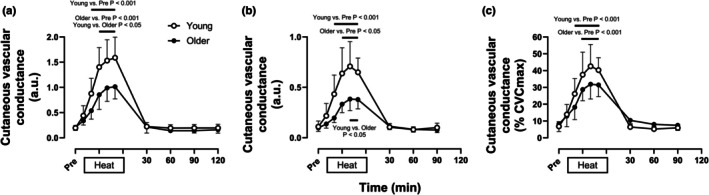
Calf cutaneous vascular conductance via integrated (a) and single point probes (b), and percent of maximal cutaneous vascular conductance via single point probes (c) at normothermic baseline (Pre), every 10 min throughout 60 min of passive heating, and every 30 min throughout normothermic post‐heating recovery in young (open circles, *n* = 16) and older individuals (filled circles, *n* = 9). Values are means ±95% confidence intervals. Due to technical challenges, cutaneous vascular conductance was not obtained in one young participant 10 and 20 minutes into heating (Panels a–c), one young participant 50 min into heating (Panels b and c), one young participant 60 min into post‐heating recovery (Panel a), and one older participant 30 min into post‐heating recovery (Panels a–c). Maximal cutaneous vascular conductance was not obtained in one older participant (Panel c: *n* = 8 older). Inferences were drawn from two‐way mixed‐effects models (age X time) using Dunnett's multiple comparisons test for changes over time versus baseline within each age group and Šidák's multiple comparisons test to compare between groups at the same time point.

As depicted in Figure 3, calf blood flow [Young: 1.7 (1.4, 2.0) mL/min/dL tissue, Older: 1.1 (0.6, 1.5) mL/min/dL tissue, *p* = 0.39] and vascular conductance [Young: 21 (17, 24) mL/min/dL tissue/mmHg, Older: 13 (7, 18) mL/min/dL tissue/mmHg, *p* = 0.45] did not differ between young and older adults at baseline. While increases in calf blood flow [Young: +3.0 (+2.3, +3.7) mL/min/dL tissue, Older: +2.0 (+1.3, +2.6) mL/min/dL tissue, *p* < 0.01] and vascular conductance [Young: +41 (+32, +50) mL/dL tissue/mmHg, Older: +23 (+15, +31) mL/dL tissue/mmHg, *p* < 0.001] were attenuated in older compared to young adults during heating, these values did not differ from baseline values throughout post‐heating recovery in either age group. As depicted in Figure 4 (Panel a), cutaneous vascular conductance assessed using the integrated laser Doppler probes was similar between age groups at baseline [Young: 0.20 (0.15, 0.24) a.u, Older: 0.21 (0.16, 0.26) a.u., *p* > 0.99], increased more with passive heating in young vs. older adults [Young: +1.39 (+1.01, +1.78) a.u., Older: +0.81 (+0.60, +1.02) a.u., *p* < 0.01], and returned to baseline values throughout post‐heating recovery in both age groups. Cutaneous vascular conductance assessed using the single point laser Doppler probes (Figure 4, Panels b and c) housed within local heating units followed a similar pattern at baseline and throughout heating and post‐heating recovery. When expressed as a percentage of maximum cutaneous vascular conductance, the increase in cutaneous vascular conductance during passive heating did not differ between age groups [Young: +33 (+28, +39)% CVCmax, Older: +30 (+13, +46)% CVCmax, *p* = 0.98].

### 3.5 Carotid baroreflex function

**FIGURE 5:**
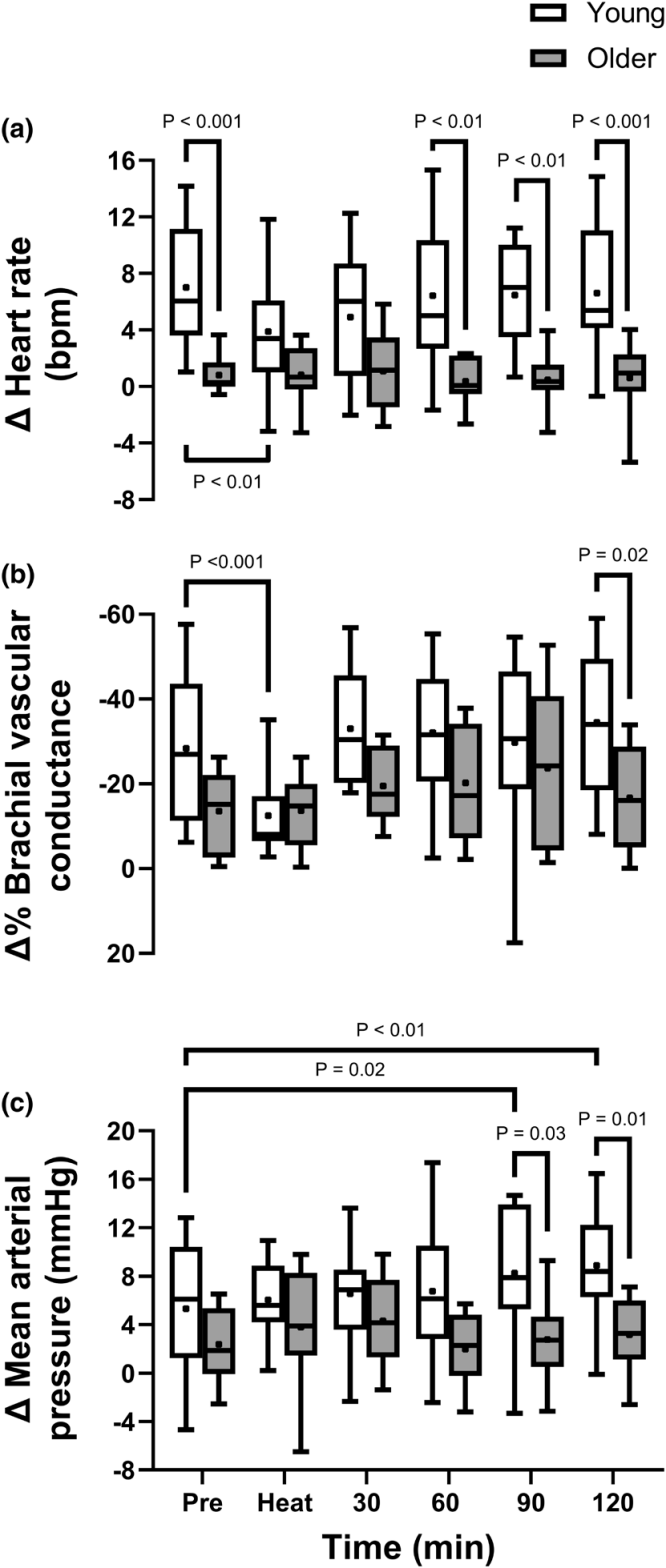
Cardiac (a), brachial vascular (b), and blood pressure (c) responses to 50 mmHg neck pressure at normothermic baseline (Pre), at the end of 60 min of passive heating, and every 30 min throughout 2 h of normothermic post‐heating recovery in young (open bars, *n* = 16) and older individuals (filled bars, *n* = 9). Each box represents the median and the 25th and 75th percentile. Whiskers represent the 5th and 95th percentiles. The symbol within each box represents the mean. Due to technical challenges, cardiac, brachial vascular, and blood pressure responses were not obtained in one older participant at 60 and 90 min into post‐heating recovery, and brachial vascular responses were not obtained in one older participant at baseline and one older participant at the end of heating. Inferences were drawn from two‐way mixed‐effects models (age X time) using Dunnett's multiple comparisons test for changes over time versus baseline within each age group and Šidák's multiple comparisons test to compare between groups at the same time point.

Table 2 displays the prevailing brachial blood flow and vascular conductance during the 3 s end‐expiratory breath‐hold immediately preceding neck pressure applications at each time point. Figure 5 displays the heart rate, brachial vascular conductance, and arterial pressure responses to simulated hypotension via applied neck pressure. Cardiac responses to neck pressure were attenuated in older compared to young adults at baseline [Young: +7 (+5, +9) bpm, Older: +1 (0, +2) bpm, *p* < 0.001], were attenuated in young but not older adults at the end of passive heating, and did not differ from baseline values throughout post‐heating recovery in either age group. The brachial vascular response to neck pressure did not differ between young and older adults at baseline [Young: −28 (−38, −19)%, Older: −14 (−22, −5)%, *p* = 0.16], was attenuated at the end of passive heating in young but not older adults, and did not differ from baseline values throughout post‐heating recovery in either age group. Arterial pressure responses to neck pressure were similar between age groups at baseline [Young: +5 (+2, +8) mmHg, Older: +2 (0, +5) mmHg, *p* = 0.49], did not differ from baseline values at the end of passive heating in either age group, and were increased relative to baseline from 90 min of post heating recovery in young, but not older individuals.

**TABLE 2:** Brachial blood flow and vascular conductance in young and older adults during passive heating and post‐heating recovery.

Variable		Baseline	End of heating	Time into post‐heating recovery			
				30 min	60 min	90 min	120 min
Brachial blood flow (mL/min)	Young	46 (30, 62)	153 (104, 203)*	46 (36, 57)	51 (35, 66)	49 (32, 66)	50 (30, 70)
	Older	42 (28, 55)	114 (70, 159)**	49 (37, 61)	43 (28, 57)	47 (30, 64)	42 (31, 54)
Brachial vascular conductance (mL/min/mmHg)	Young	0.53 (0.35, 0.72)	2.25 (1.52, 2.98)*	0.59 (0.44, 0.73)	0.59 (0.40, 0.78)	0.57 (0.37, 0.76)	0.55 (0.35, 0.75)
	Older	0.48 (0.36, 0.60)	1.84 (1.06, 2.61)**	0.63 (0.44, 0.81)	0.48 (0.32, 0.64)	0.49 (0.32, 0.65)	0.43 (0.34, 0.52)

*Note*: Brachial blood flow and vascular conductance at baseline, at the end of passive heating, and every 30 min throughout 2 h of normothermic recovery in young (*n* = 16) and older individuals (*n* = 9). Values are means ± 95% confidence intervals. Due to technical challenges, data were not obtained in one older participant at 60 and 90 min into post‐heating recovery. Inferences were drawn from two‐way mixed‐effects models (age X time) using Dunnett's multiple comparisons test for changes over time versus baseline within each age group and Šidák's multiple comparisons test to compare between groups at the same time point.

* *p* < 0.05 versus baseline within young.

** *p* < 0.05 versus baseline within older.

## 4 DISCUSSION

This study aimed to characterize and compare the systemic cardiovascular and carotid baroreflex support of arterial pressure during recovery from whole‐body, passive heating in normotensive young and older adults. Contrary to our hypothesis, recovery from a single session of whole‐body, passive heat stress did not promote a sustained reduction in arterial pressure or “post‐heating hypotension” or sustained alterations in systemic cardiovascular and carotid baroreflex support of arterial pressure in young or older adults.

### 4.1 Influence of age on acute responses to heat stress

Passive heating increased skin, core, and mean body temperature similarly between age groups. Although we noted less of an increase in calf vascular conductance and cutaneous vascular conductance during heating in older versus young adults, young and older adults displayed similar increases in systemic vascular conductance and cardiac output during passive heating. In contrast, the majority of previous studies have noted that heating‐induced increases in cardiac output are reduced by ~50% in older versus young adults (Gagnon et al., 2016, 2017; Greaney et al., 2015; Minson et al., 1998). This discrepancy could result from differences in the characteristics of the older adults studied. Indeed, among a cohort of physically active older adults similar to that of the present study, Lucas et al. (2015) found that young and older adults demonstrated similar increases in cardiac output (Δ cardiac output young: 1.9 L/min, older: 1.5 L/min) during whole‐body, passive heating (Δ intestinal temperature ~1°C).

Increases in cardiac output with heating were mediated differently between age groups. In young adults, increases in cardiac output were supported by increases in heart rate amid reductions in stroke volume. In contrast, older adults tended to display attenuated increases in heart rate during passive heating compared to young adults, but stroke volume was well maintained. Previous studies have noted diminished increases in heart rate with heating among older versus young adults (Gagnon et al., 2016, 2017; Greaney et al., 2015; Lucas et al., 2015). In contrast with the present study, stroke volume is typically well maintained or slightly increased during passive heating in young adults (Brothers et al., 2009; Bundgaard‐Nielsen et al., 2010; Wilson et al., 2009; Wilson & Crandall, 2011). Furthermore, the ability to maintain or increase stroke volume during passive heat stress is typically either preserved (Gagnon et al., 2016, 2017; Greaney et al., 2015; Lucas et al., 2015) or reduced (Fischer et al., 2022; Minson et al., 1998) with advancing age. As the inotropic response to heating was not likely reduced among young adults, the reduction in stroke volume observed during heating may reflect a reduction in central venous pressure due to greater peripheral displacement of blood volume or greater intravascular volume loss secondary to exaggerated sweat losses. Although we did not directly assess sweat rate or changes in blood or plasma volume, young and older adults were matched for hydration status (urine‐specific gravity Young: 1.016 (1.011, 1.021), Older: 1.017 (1.011, 1.023), *p* = 0.86), provided the same amount of water to drink during heating (3 mL/kg body weight; Young: 208 (184, 231) mL, Older: 215 (170, 261) mL, *p* = 0.71), and had similar estimated sweat losses.

These central and peripheral cardiovascular responses culminated in an ~7 mmHg reduction in mean arterial pressure during heating among young adults due to a pronounced reduction in diastolic blood pressure, while mean arterial pressure was well maintained in older adults during heating.

### 4.2 Central and peripheral hemodynamics during post‐heating recovery

Skin temperature returned to baseline levels upon the re‐circulation of 34°C water during post‐heating recovery, but elevations in core temperature persisted 90 min into post‐heating recovery in both age groups. While the study provided no evidence of a sustained reduction in arterial pressure or “post‐heating hypotension” during recovery from whole‐body, passive heat stress, previous studies have yielded conflicting findings and noted that mean arterial pressure is either increased (Hemingway et al., 2023), decreased (Engelland et al., 2020b; Francisco et al., 2021; Laukkanen et al., 2018; Neff et al., 2016; Thomas et al., 2017), or unchanged (Engelland et al., 2020a; Gravel et al., 2019; Richey et al., 2022) during recovery from passive heating. The elevations in arterial pressure observed with extended recovery in older adults may reflect the confounding influence of an increased level of participant arousal due to the many measurements or the circadian blood pressure rhythm as, on average, baseline blood pressures were conducted between ~1000 and 1100 and post‐heating recovery began ~1230.

Cardiac output did not differ from baseline levels throughout post‐heating recovery. While Romero et al. demonstrated sustained elevations in cardiac output at 30 min post‐heating recovery in young adults (Romero et al., 2017), other studies have demonstrated a return of cardiac output to baseline values within 30 min of post‐heating recovery in young and older adults (Engelland et al., 2020b; Francisco et al., 2021; Romero et al., 2017). At 30 min of post‐heating recovery, cardiac output was maintained in young adults by a slightly, although not significantly, elevated heart rate as stroke volume remained below baseline levels. In contrast, heart rate and stroke volume did not differ from baseline levels throughout post‐heating recovery in older adults. The present study also provided no evidence of sustained vasodilation during post‐heating recovery as systemic vascular conductance, calf vascular conductance, and cutaneous vascular conductance did not differ from baseline levels throughout post‐heating recovery in either age group. Francisco et al. (2021) similarly noted that brachial and femoral blood flow and vascular conductance did not differ from baseline levels after 40 min of recovery following hot water immersion.

### 4.3 Carotid baroreflex function

While several studies have interrogated baroreflex regulation of arterial pressure during whole‐body passive heat stress (Crandall, 2000; Cui et al., 2002a, 2004; Keller et al., 2006; Krnjajic et al., 2016; Yamazaki et al., 1997), no study to our knowledge has evaluated baroreflex function in the post‐heating recovery period. We utilized the neck pressure technique to evaluate carotid baroreflex control of the heart, peripheral vasculature, and integrated support of arterial pressure at baseline and during passive heating and post‐heating recovery. Older adults displayed attenuated cardiac responses to simulated hypotension compared to young adults at baseline, although vascular and integrated carotid baroreflex support of arterial pressure did not differ between age groups at baseline. Numerous studies have similarly demonstrated reductions in carotid baroreflex function with advancing age.

The cardiac and vascular responses to neck pressure were attenuated in young but not older adults during passive heating. Similarly, some (Crandall, 2000; Yamazaki et al., 1997), but not all (Krnjajic et al., 2016), studies in young individuals have noted that although heat stress does not alter the maximum gain of the carotid‐cardiac baroreflex, it shifts the operating point of the reflex closer to the maximum response threshold, effectively reducing the capacity to increase heart rate in response to hypotension. Scremin and Kenney (2004) previously evaluated the forearm vascular conductance response to lower body negative pressure among young and older men in normothermic and heat stressed (Δ sublingual temperature 0.9°C) conditions. Although vascular responses between thermal conditions were not directly compared, lower body negative pressure (−30 mmHg) reduced forearm vascular conductance by ~47% in young and ~33% in older men under thermoneutral conditions and by ~42% in young and ~19% in older men during heat stress. These trends support our observations of attenuated brachial vascular responses to carotid baroreflex unloading during passive heating versus normothermia among young adults. However, in contrast to our findings, the heating‐induced attenuation of vascular responses to baroreceptor unloading was also present among the older men studied by Scremin and Kenney.

Although the present study noted a differential impact of passive heating on the brachial vascular responses to carotid baroreceptor unloading between age groups, the brachial vascular responses to carotid baroreceptor unloading returned to baseline levels throughout post‐heating recovery in both age groups. Engelland et al. similarly demonstrated that sympathetic vascular transduction, assessed as the relation between sympathetic nerve activity and leg vascular conductance during isometric handgrip, did not differ from baseline levels 30 min following leg heating in young and older adults (Engelland et al., 2020b).

In both age groups, passive heating and the first 90 min of post‐heating recovery did not alter the arterial pressure responses to neck pressure. The preservation of arterial pressure responses to neck pressure during passive heating among young adults was somewhat surprising given the simultaneously attenuated cardiac and brachial vascular responses and could reflect maintained or enhanced vascular responsiveness within other circulations (e.g., splanchnic or renal). Similarly, Krnjajic et al. (2016) reported that passive, whole‐body heating (Δ core temperature ~1°C via telemetric pill) did not alter the maximum gain of carotid baroreflex control of arterial pressure or the peak change in mean arterial pressure across varying levels of 5 s applications of neck pressure in young men (+15–45 mmHg).

### 4.4 Limitations

Although the neck pressure technique allowed for characterization of the dynamic changes in cardiac, peripheral vascular, and integrated carotid baroreflex responsiveness during heating and post‐heating recovery, several considerations warrant mention. First, as baroreflex mediated responses are often asymmetrical between falling and rising pressure stimuli (Eckberg, 1980), our limited assessment of cardiac, vascular, and blood pressure responses to neck pressure (simulated hypotension) may not adequately describe baroreflex responses to rising pressure stimuli. Moreover, as we did not utilize various neck pressure/suction combinations, our results cannot be used to derive parameters describing baroreflex function across its entire operating range. Due to challenges in recruiting exceptionally healthy older adults (no medications, blood pressure ≤140/90 mmHg, body mass index >18.5 and <35 kg·m^−2^) who were not endurance training, we present data from more young (*n* = 16) versus older adults (*n* = 9). Additionally, while men and women were equally represented within the younger age group, more women (*n* = 6) than men (*n* = 3) comprised the older age group. Therefore, differences noted between age groups may be unduly influenced by the underrepresentation of older versus young adults or men among the older cohort.

### 4.5 Experimental considerations

We were adequately powered (1 – *β* >0.80) to detect a 6 mmHg change in mean arterial pressure (30 min post‐heating recovery vs. baseline) in young (*n* = 16, SD 8 mmHg) and older individuals (*n* = 9, SD 5 mmHg), and an 8.2 mmHg difference in the change in mean arterial pressure between groups, similar to what we (Francisco et al., 2021) and others (Engelland et al., 2020b; Laukkanen et al., 2018; Neff et al., 2016; Romero et al., 2017; Thomas et al., 2017) have reported. Therefore, it is possible that an aspect of the overall study design (heating or recovery protocol, participant characteristics) was not optimal in promoting post‐heating hypotension. For example, although post‐heating hypotension has been demonstrated following supine heating via water‐perfused suit (Engelland et al., 2020b), water immersion may be a critical factor in promoting post‐heating hypotension and/or sustained vasodilation. Indeed, several previous studies that have observed post‐heating hypotension and sustained vasodilation have utilized a seated hot water immersion heating modality (Francisco et al., 2021; Romero et al., 2017; Thomas et al., 2017), and Roxburgh et al. (2025) recently demonstrated that 20–40 min of immersion in hot (40°C) or thermoneutral water (36.5°C) reduced 24‐hour systolic blood pressure by 6–7 mmHg among hypertensive individuals. Alternatively, skin temperature and/or blood flow during post‐heating recovery may be a critical factor in promoting post‐heating hypotension and/or sustained vasodilation. This study facilitated recovery by perfusing 34°C water through a water‐perfused suit upon the cessation of heating which may have promoted the prompt return of skin temperature, skin and systemic vascular conductance, and arterial pressure to baseline levels. Although previous studies using this “active” skin cooling approach have elicited post‐heating hypotension (Engelland et al., 2020b), other studies that have noted post‐heating hypotension and sustained elevations of skin and/or limb blood flow and systemic vascular conductance have utilized a more “passive” cooling approach (recovery in an ~22–24°C room) (Francisco et al., 2021; Romero et al., 2017; Thomas et al., 2017). Finally, both young and older individuals in the present study were normotensive and post‐heating hypotension, like post‐exercise hypotension, may be more consistent and/or pronounced among hypertensive (Kenney & Seals, 1993).

### 4.6 Perspectives and significance

This study presents novel data displaying the time course of thermal, systemic cardiovascular, and neurovascular recovery from whole‐body, passive heat stress in young and older adults. We demonstrated that the arterial pressure, systemic cardiovascular, and baroreflex responses that accompanied acute heat stress were transient and did not persist beyond 30 min of post‐heating recovery in young or older individuals despite continued elevations in core temperature. While the heating and recovery paradigms employed in this study did not successfully elicit post‐heating hypotension, future studies examining the influence of heating modality, recovery, or participant characteristics on the expression of the post‐heating recovery profile are warranted. In the same way that we have defined the F.I.T.T. (Frequency, Intensity, Time, Type) principles with respect to maximizing the cardiovascular benefits of exercise training, it is unclear whether an optimal heating regimen exists that maximizes the cardiovascular benefits of chronic heat therapy (Brunt & Minson, 2021; Cheng & MacDonald, 2019). An improved understanding of the post‐heating recovery period and the factors that modify it are important considerations in addressing this question. Alternatively, if acute heat stress does not consistently yield a post‐stress hypotensive response, this information should inform the clinical utility of heat therapy as a cardiovascular therapeutic option.

## AUTHOR CONTRIBUTIONS

E.A.L., C.T.M., and J.R.H. conceived of and designed the experiment. E.A.L., B.W.K., E.L.R., B.M.G., and K.S.S.A. performed experiments. E.A.L. analyzed data and prepared figures. E.A.L., C.T.M., and J.R.H. interpreted results of experiments. E.A.L., C.T.M., and J.R.H. drafted the manuscript. E.A.L., B.W.K., E.L.R., B.M.G., and K.S.S.A., W.L.K., C.T.M., and J.R.H. edited and revised the manuscript for intellectual content. All authors approved the final version of the manuscript and agreed to be accountable for all aspects of the work. All persons designated as authors qualify for authorship, and all those who qualify for authorship are listed.

## FUNDING INFORMATION

This work was supported by the National Institutes of Health (NHLBI) grants HL144128 and HL158087 (F31‐Larson), the Eugene and Clarissa Evonuk Memorial Graduate Fellowship (Larson), and the Kenneth and Kenda Singer Endowed Professorship in Human Physiology (Minson).

## CONFLICT OF INTEREST STATEMENT

No conflicts of interest, financial or otherwise, are declared by the authors.

## Data Availability

Data will be made available upon reasonable request to the corresponding author.

## References

[phy270554-bib-0001] Brothers, R. M. , Bhella, P. S. , Shibata, S. , Wingo, J. E. , Levine, B. D. , & Crandall, C. G. (2009). Cardiac systolic and diastolic function during whole body heat stress. American Journal of Physiology. Heart and Circulatory Physiology, 296, H1150–H1156. 19218504 10.1152/ajpheart.01069.2008 PMC2670696

[phy270554-bib-0002] Brunt, V. E. , & Minson, C. T. (2021). Heat therapy: Mechanistic underpinnings and applications to cardiovascular health. Journal of Applied Physiology, 130, 1684–1704. 33792402 10.1152/japplphysiol.00141.2020 PMC8285605

[phy270554-bib-0003] Buck, T. M. , Romero, S. A. , Ely, M. R. , Sieck, D. C. , Abdala, P. M. , & Halliwill, J. R. (2015). Neurovascular control following small muscle‐mass exercise in humans. Physiological Reports, 3, e12289. 25649250 10.14814/phy2.12289 PMC4393198

[phy270554-bib-0004] Buckwalter, J. B. , & Clifford, P. S. (2001). The paradox of sympathetic vasoconstriction in exercising skeletal muscle. Exercise and Sport Sciences Reviews, 29, 159–163. 11688788 10.1097/00003677-200110000-00005

[phy270554-bib-0005] Bundgaard‐Nielsen, M. , Wilson, T. E. , Seifert, T. , Secher, N. H. , & Crandall, C. G. (2010). Effect of volume loading on the frank‐Starling relation during reductions in central blood volume in heat‐stressed humans. The Journal of Physiology, 588, 3333–3339. 20603336 10.1113/jphysiol.2010.191981 PMC2976026

[phy270554-bib-0006] Carpio‐Rivera, E. , Moncada‐Jiménez, J. , Salazar‐Rojas, W. , & Solera‐Herrera, A. (2016). Acute effects of exercise on blood pressure: A meta‐analytic investigation. Arquivos Brasileiros de Cardiologia. 10.5935/abc.20160064 PMC4914008 27168471

[phy270554-bib-0007] Cheng, J. L. , & MacDonald, M. J. (2019). Effect of heat stress on vascular outcomes in humans. Journal of Applied Physiology, 126, 771–781. 30676869 10.1152/japplphysiol.00682.2018 PMC6459390

[phy270554-bib-0008] Crandall, C. G. (2000). Carotid baroreflex responsiveness in heat‐stressed humans. American Journal of Physiology. Heart and Circulatory Physiology, 279, H1955–H1962. 11009485 10.1152/ajpheart.2000.279.4.H1955

[phy270554-bib-0009] Cui, J. , Wilson, T. E. , & Crandall, C. G. (2002a). Baroreflex modulation of sympathetic nerve activity to muscle in heat‐stressed humans. American Journal of Physiology. Regulatory, Integrative and Comparative Physiology, 282, R252–R258. 11742845 10.1152/ajpregu.00337.2001

[phy270554-bib-0010] Cui, J. , Wilson, T. E. , & Crandall, C. G. (2002b). Phenylephrine‐induced elevations in arterial blood pressure are attenuated in heat‐stressed humans. American Journal of Physiology. Regulatory, Integrative and Comparative Physiology, 283, R1221–R1226. 12376416 10.1152/ajpregu.00195.2002

[phy270554-bib-0011] Cui, J. , Wilson, T. E. , & Crandall, C. G. (2004). Muscle sympathetic nerve activity during lower body negative pressure is accentuated in heat‐stressed humans. Journal of Applied Physiology, 96, 2103–2108. 14978004 10.1152/japplphysiol.00717.2003

[phy270554-bib-0012] Eckberg, D. L. (1980). Nonlinearities of the human carotid baroreceptor‐cardiac reflex. Circulation Research, 47, 208–216. 7397953 10.1161/01.res.47.2.208

[phy270554-bib-0013] Eckberg, D. L. , Kifle, Y. T. , & Roberts, V. L. (1980). Phase relationship between normal human respiration and baroreflex responsiveness. The Journal of Physiology, 304, 489–502. 7441548 10.1113/jphysiol.1980.sp013338 PMC1282944

[phy270554-bib-0014] Engelland, R. E. , Hemingway, H. W. , Tomasco, O. G. , OLIVENCIA‐Yurvati, A. H. , & Romero, S. A. (2020a). Acute lower leg hot water immersion protects macrovascular dilator function following ischaemia‐reperfusion injury in humans. Experimental Physiology, 105, 302–311. 31707732 10.1113/EP088154 PMC7429992

[phy270554-bib-0015] Engelland, R. E. , Hemingway, H. W. , Tomasco, O. G. , OLIVENCIA‐Yurvati, A. H. , & Romero, S. A. (2020b). Neural control of blood pressure is altered following isolated leg heating in aged humans. American Journal of Physiology‐Heart and Circulatory Physiology, 318, H976–H984. 32142377 10.1152/ajpheart.00019.2020 PMC7191488

[phy270554-bib-0016] Fischer, M. , Moralez, G. , Sarma, S. , Macnamara, J. P. , Cramer, M. N. , Huang, M. , Romero, S. A. , Hieda, M. , Shibasaki, M. , Ogoh, S. , & Crandall, C. G. (2022). Altered cardiac beta1 responsiveness in hyperthermic older adults. American Journal of Physiology‐Regulatory, Integrative and Comparative Physiology, 323, R581–R588. 36094450 10.1152/ajpregu.00040.2022 PMC9602700

[phy270554-bib-0017] Fisher, J. P. , Kim, A. , Young, C. N. , Ogoh, S. , Raven, P. B. , Secher, N. H. , & Fadel, P. J. (2009). Influence of ageing on carotid baroreflex peak response latency in humans. The Journal of Physiology, 587, 5427–5439. 19805748 10.1113/jphysiol.2009.177998 PMC2793874

[phy270554-bib-0018] Fitzgerald, W. (1981). Labile hypertension and jogging: New diagnostic tool or spurious discovery? British Medical Journal (Clinical Research Ed.), 282, 542–544. 6780119 10.1136/bmj.282.6263.542 PMC1504300

[phy270554-bib-0019] Francisco, M. A. , Colbert, C. , Larson, E. A. , Sieck, D. C. , Halliwill, J. R. , & Minson, C. T. (2021). Hemodynamics of post‐exercise vs. post hot water immersion recovery. Journal of Applied Physiology, 130, 1362–1372. 33630675 10.1152/japplphysiol.00260.2020 PMC8354820

[phy270554-bib-0020] Gagnon, D. , Romero, S. A. , Ngo, H. , Sarma, S. , Cornwell, W. K., 3rd , Poh, P. Y. , Stoller, D. , Levine, B. D. , & Crandall, C. G. (2016). Healthy aging does not compromise the augmentation of cardiac function during heat stress. Journal of Applied Physiology, 121, 885–892. 27609201 10.1152/japplphysiol.00643.2016 PMC5142306

[phy270554-bib-0021] Gagnon, D. , Romero, S. A. , Ngo, H. , Sarma, S. , Cornwell, W. K. R. , Poh, P. Y. S. , Stoller, D. , Levine, B. D. , & Crandall, C. G. (2017). Volume loading augments cutaneous vasodilatation and cardiac output of heat stressed older adults. The Journal of Physiology, 595, 6489–6498. 28833129 10.1113/JP274742 PMC5638885

[phy270554-bib-0022] Gagnon, D. , Schlader, Z. J. , & Crandall, C. G. (2015). Sympathetic activity during passive heat stress in healthy aged humans. The Journal of Physiology, 593, 2225–2235. 25752842 10.1113/JP270162 PMC4422574

[phy270554-bib-0023] Gravel, H. , Coombs, G. B. , Behzadi, P. , MARCOUX‐Clement, V. , Barry, H. , Juneau, M. , Nigam, A. , & Gagnon, D. (2019). Acute effect of Finnish sauna bathing on brachial artery flow‐mediated dilation and reactive hyperemia in healthy middle‐aged and older adults. Physiological Reports, 7, e14166. 31293098 10.14814/phy2.14166 PMC6640592

[phy270554-bib-0024] Greaney, J. L. , Stanhewicz, A. E. , Proctor, D. N. , Alexander, L. M. , & Kenney, W. L. (2015). Impairments in central cardiovascular function contribute to attenuated reflex vasodilation in aged skin. Journal of Applied Physiology, 119, 1411–1420. 26494450 10.1152/japplphysiol.00729.2015 PMC4683344

[phy270554-bib-0025] Halliwill, J. R. (2001). Mechanisms and clinical implications of post‐exercise hypotension in humans. Exercise and Sport Sciences Reviews, 29, 65–70. 11337825 10.1097/00003677-200104000-00005

[phy270554-bib-0026] Halliwill, J. R. , Buck, T. M. , Lacewell, A. N. , & Romero, S. A. (2013). Postexercise hypotension and sustained postexercise vasodilatation: What happens after we exercise? Experimental Physiology, 98, 7–18. 22872658 10.1113/expphysiol.2011.058065

[phy270554-bib-0027] Halliwill, J. R. , Taylor, J. A. , & Eckberg, D. L. (1996). Impaired sympathetic vascular regulation in humans after acute dynamic exercise. The Journal of Physiology, 495(Pt 1), 279–288. 8866370 10.1113/jphysiol.1996.sp021592 PMC1160743

[phy270554-bib-0028] Hemingway, H. W. , Richey, R. E. , Moore, A. M. , Saul, B. M. , Shokraeifard, A. M. , Cope, H. L. , OLIVENCIA‐Yurvati, A. H. , Cunningham, R. L. , Smith, M. L. , & Romero, S. A. (2023). Effect of acute heat exposure on the pressor response to a voluntary hypoxic apnea. Journal of Applied Physiology, 135, 542–548. 37439242 10.1152/japplphysiol.00245.2023 PMC10538993

[phy270554-bib-0029] Jackson, A. S. , & Pollock, M. L. (1978). Generalized equations for predicting body density of men. The British Journal of Nutrition, 40, 497–504. 718832 10.1079/bjn19780152

[phy270554-bib-0030] Jackson, A. S. , Pollock, M. L. , & Ward, A. (1980). Generalized equations for predicting body density of women. Medicine and Science in Sports and Exercise, 12, 175–181. 7402053

[phy270554-bib-0031] Johnson, B. D. , Beck, K. C. , Proctor, D. N. , Miller, J. , Dietz, N. M. , & Joyner, M. J. (2000). Cardiac output during exercise by the open circuit acetylene washin method: Comparison with direct Fick. Journal of Applied Physiology, 88, 1650–1658. 10797126 10.1152/jappl.2000.88.5.1650

[phy270554-bib-0032] Keller, D. M. , Cui, J. , Davis, S. L. , Low, D. A. , & Crandall, C. G. (2006). Heat stress enhances arterial baroreflex control of muscle sympathetic nerve activity via increased sensitivity of burst gating, not burst area, in humans. The Journal of Physiology, 573, 445–451. 16581857 10.1113/jphysiol.2006.108662 PMC1779723

[phy270554-bib-0033] Keller, D. M. , Fadel, P. J. , Ogoh, S. , Brothers, R. M. , Hawkins, M. , OLIVENCIA‐Yurvati, A. , & Raven, P. B. (2004). Carotid baroreflex control of leg vasculature in exercising and non‐exercising skeletal muscle in humans. The Journal of Physiology, 561, 283–293. 15388778 10.1113/jphysiol.2004.071944 PMC1665330

[phy270554-bib-0034] Keller, D. M. , Wasmund, W. L. , Wray, D. W. , Ogoh, S. , Fadel, P. J. , Smith, M. L. , & Raven, P. B. (2003). Carotid baroreflex control of leg vascular conductance at rest and during exercise. Journal of Applied Physiology, 94, 542–548. 12391067 10.1152/japplphysiol.00817.2002

[phy270554-bib-0035] Kenney, M. J. , & Seals, D. R. (1993). Postexercise hypotension. Key features, mechanisms, and clinical significance. Hypertension, 22, 653–664. 8225525 10.1161/01.hyp.22.5.653

[phy270554-bib-0036] Krnjajic, D. , Allen, D. R. , Butts, C. L. , & Keller, D. M. (2016). Carotid baroreflex control of heart rate is enhanced, while control of mean arterial pressure is preserved during whole body heat stress in young healthy men. American Journal of Physiology. Regulatory, Integrative and Comparative Physiology, 311, R735–R741. 27488886 10.1152/ajpregu.00152.2016

[phy270554-bib-0037] Larson, E. A. (2023). Systemic Cardiovascular and Carotid Baroreflex Support of Blood Pressure during Recovery from Passive Heat Stress in Young and Older Adults. University of Oregon.10.14814/phy2.7055440930837

[phy270554-bib-0038] Laukkanen, T. , Kunutsor, S. K. , Zaccardi, F. , Lee, E. , Willeit, P. , Khan, H. , & Laukkanen, J. A. (2018). Acute effects of sauna bathing on cardiovascular function. Journal of Human Hypertension, 32, 129–138. 29269746 10.1038/s41371-017-0008-z

[phy270554-bib-0039] Lucas, R. A. , Sarma, S. , Schlader, Z. J. , Pearson, J. , & Crandall, C. G. (2015). Age‐related changes to cardiac systolic and diastolic function during whole‐body passive hyperthermia. Experimental Physiology, 100, 422–434. 25641368 10.1113/expphysiol.2014.083014 PMC4948727

[phy270554-bib-0040] McCord, J. L. , & Halliwill, J. R. (2006). H1 and H2 receptors mediate postexercise hyperemia in sedentary and endurance exercise‐trained men and women. Journal of Applied Physiology, 101, 1693–1701. 16888049 10.1152/japplphysiol.00441.2006

[phy270554-bib-0041] Minson, C. T. (2010). Thermal provocation to evaluate microvascular reactivity in human skin. Journal of Applied Physiology, 109, 1239–1246. 20507974 10.1152/japplphysiol.00414.2010 PMC2963329

[phy270554-bib-0042] Minson, C. T. , Wladkowski, S. L. , Cardell, A. F. , Pawelczyk, J. A. , & Kenney, W. L. (1998). Age alters the cardiovascular response to direct passive heating. Journal of Applied Physiology, 84, 1323–1332. 9516200 10.1152/jappl.1998.84.4.1323

[phy270554-bib-0043] Minson, C. T. , Wladkowski, S. L. , Pawelczyk, J. A. , & Kenney, W. L. (1999). Age, splanchnic vasoconstriction, and heat stress during tilting. The American Journal of Physiology, 276, R203–R212. 9887196 10.1152/ajpregu.1999.276.1.r203

[phy270554-bib-0044] Neff, D. , Kuhlenhoelter, A. M. , Lin, C. , Wong, B. J. , Motaganahalli, R. L. , & Roseguini, B. T. (2016). Thermotherapy reduces blood pressure and circulating endothelin‐1 concentration and enhances leg blood flow in patients with symptomatic peripheral artery disease. American Journal of Physiology. Regulatory, Integrative and Comparative Physiology, 311, R392–R400. 27335279 10.1152/ajpregu.00147.2016 PMC5008667

[phy270554-bib-0045] Pellinger, T. K. , & Halliwill, J. R. (2007). Effect of propranolol on sympathetically mediated leg vasoconstriction in humans. The Journal of Physiology, 583, 797–809. 17627989 10.1113/jphysiol.2007.137422 PMC2277027

[phy270554-bib-0046] Richey, R. E. , Hemingway, H. W. , Moore, A. M. , OLIVENCIA‐Yurvati, A. H. , & Romero, S. A. (2022). Acute heat exposure improves microvascular function in skeletal muscle of aged adults. American Journal of Physiology‐Heart and Circulatory Physiology, 322, H386–H393. 35060753 10.1152/ajpheart.00645.2021 PMC8858667

[phy270554-bib-0047] Romero, S. A. , Gagnon, D. , Adams, A. N. , Cramer, M. N. , Kouda, K. , & Crandall, C. G. (2017). Acute limb heating improves macro‐ and microvascular dilator function in the leg of aged humans. American Journal of Physiology. Heart and Circulatory Physiology, 312, H89–H97. 27836894 10.1152/ajpheart.00519.2016 PMC5283915

[phy270554-bib-0048] Roxburgh, B. H. , Campbell, H. A. , Cotter, J. D. , Williams, M. J. A. , & Thomas, K. N. (2025). Both hot‐ and thermoneutral‐water immersion reduce 24‐h blood pressure in people with hypertension: A randomized crossover study. Temperature (Austin), 12, 166–178. 40330610 10.1080/23328940.2025.2465025 PMC12051523

[phy270554-bib-0049] Scremin, G. , & Kenney, W. L. (2004). Aging and the skin blood flow response to the unloading of baroreceptors during heat and cold stress. Journal of Applied Physiology, 96, 1019–1025. 14594858 10.1152/japplphysiol.00928.2003

[phy270554-bib-0050] Thomas, K. N. , VAN Rij, A. M. , Lucas, S. J. , & Cotter, J. D. (2017). Lower‐limb hot‐water immersion acutely induces beneficial hemodynamic and cardiovascular responses in peripheral arterial disease and healthy, elderly controls. American Journal of Physiology‐Regulatory, Integrative and Comparative Physiology, 312, R281–R291. 28003211 10.1152/ajpregu.00404.2016

[phy270554-bib-0051] Whelton, P. K. , Carey, R. M. , Aronow, W. S. , Casey, D. E., Jr. , Collins, K. J. , Dennison Himmelfarb, C. , Depalma, S. M. , Gidding, S. , Jamerson, K. A. , Jones, D. W. , Maclaughlin, E. J. , Muntner, P. , Ovbiagele, B. , Smith, S. C., Jr. , Spencer, C. C. , Stafford, R. S. , Taler, S. J. , Thomas, R. J. , Williams, K. A. , … Wright, J. T., Jr. (2018). 2017 ACC/AHA/AAPA/ABC/ACPM/AGS/APhA/ASH/ASPC/NMA/PCNA guideline for the prevention, detection, evaluation, and management of high blood pressure in adults: A report of the American College of Cardiology/American Heart Association task force on clinical practice guidelines. Hypertension, 71, e13–e115. 29133356 10.1161/HYP.0000000000000065

[phy270554-bib-0052] Wilson, T. E. , Brothers, R. M. , Tollund, C. , Dawson, E. A. , Nissen, P. , Yoshiga, C. C. , Jons, C. , Secher, N. H. , & Crandall, C. G. (2009). Effect of thermal stress on frank‐Starling relations in humans. The Journal of Physiology, 587, 3383–3392. 19417092 10.1113/jphysiol.2009.170381 PMC2727045

[phy270554-bib-0053] Wilson, T. E. , & Crandall, C. G. (2011). Effect of thermal stress on cardiac function. Exercise and Sport Sciences Reviews, 39, 12–17. 21088607 10.1097/JES.0b013e318201eed6 PMC3076691

[phy270554-bib-0054] Yamazaki, F. , Sagawa, S. , Torii, R. , Endo, Y. , & Shiraki, K. (1997). Effects of acute hyperthermia on the carotid baroreflex control of heart rate in humans. International Journal of Biometeorology, 40, 200–205. 9225596 10.1007/s004840050042

